# Development of a risk prediction model for adhesive intestinal obstruction following laparoscopic surgery for acute appendicitis based on clinical characteristics and laboratory indicators

**DOI:** 10.3389/fmed.2026.1724502

**Published:** 2026-05-01

**Authors:** Jie Ji, Ying Wang, Yine Wang, Lei Li, Bingmei Zhou, Jiajia Guan

**Affiliations:** 1 Department of Emergency Surgery, The First Affiliated Hospital of Bengbu Medical University, Bengbu, Anhui, China; 2 Department of Gastrointestinal Surgery, The First Affiliated Hospital of Bengbu Medical University, Bengbu, Anhui, China

**Keywords:** acute appendicitis, adhesive intestinal obstruction, clinical characteristics, laboratory indicators, laparoscopic appendectomy, risk prediction model

## Abstract

**Background:**

Adhesive intestinal obstruction (AIO) is a recognized complication following laparoscopic surgery for acute appendicitis (AA), although its overall incidence remains relatively low. This study aimed to develop a risk prediction model based on clinical characteristics and laboratory indicators to facilitate early identification and prevention of postoperative AIO.

**Methods:**

In this retrospective cohort study, 298 AA patients who underwent laparoscopic surgery between January 2020 and November 2024 were enrolled. Patients were categorized into AIO (*n* = 32) and non-AIO (*n* = 266) groups. Data were collected from electronic medical records and telephone follow-ups. Univariate analysis compared clinical and laboratory variables between groups. Significant variables underwent collinearity analysis, and those without collinearity were entered into binary logistic regression to identify independent predictors. A nomogram prediction model was constructed and internally validated using bootstrap resampling. Model performance was assessed using calibration curves and the area under the receiver operating characteristic curve (AUC).

**Results:**

Compared with the non-AIO group, the AIO group exhibited significantly longer disease duration, a higher prevalence of systemic inflammatory response syndrome (SIRS), elevated white blood cell count (WBC), increased neutrophil-to-lymphocyte ratio (NLR), and lower albumin (ALB) levels (*p* < 0.05). Logistic regression identified disease duration, SIRS, WBC, and NLR as independent risk factors for AIO (OR >1, *p* < 0.05), while ALB served as a protective factor (OR <1, *p* < 0.05). The resulting nomogram demonstrated excellent discrimination, with an AUC of 0.960 (95% CI: 0.912–1.000), and the calibration curve closely aligned with the ideal standard.

**Conclusion:**

Disease duration, SIRS, WBC, NLR, and ALB are significantly associated with AIO occurrence after laparoscopic appendectomy. The developed prediction model effectively stratifies postoperative AIO risk in AA patients, potentially assisting clinicians in implementing timely preventive measures and reducing AIO incidence.

## 1 Introduction

Acute appendicitis (AA) is an acute inflammation of the appendix caused by bacterial infection and other factors. It is one of the most common acute abdominal conditions in emergency surgery and has a high incidence rate worldwide (1). According to relevant data, AA accounts for approximately 10% of all surgical emergency patients and 20 to 30% of all acute abdominal patients (2). Additionally, studies have shown that AA can occur at any age, with the highest incidence rate among young adults aged 20–30 years, accounting for approximately 40%, and a higher prevalence in males than females, with a ratio of approximately 2–3:1 (3). This condition is often caused by physical obstruction, such as fecal stones blocking the appendix lumen or abnormal swelling of lymphoid tissue obstructing the lumen (4). When the duct is blocked, bacteria proliferate internally, causing the appendix wall to become congested and swollen, resulting in severe pain. Initially, there may be mild pain in the upper abdomen or around the navel, which shifts to persistent pain in the lower right abdomen within a few hours, often accompanied by nausea, vomiting, loss of appetite, and low-grade fever (5). If timely and appropriate treatment is not administered, the swollen appendix may perforate, leading to more severe abdominal infection (6). With the continuous development of laparoscopic technology, laparoscopic appendectomy (LA) has become the preferred surgical method for treating AA due to its advantages of minimal trauma, rapid recovery, and fewer postoperative complications (7). However, despite the many advantages of LA, postoperative complications may still occur. Among these, postoperative adhesive intestinal obstruction (AIO) is a recognized albeit relatively uncommon complication, which may cause symptoms such as abdominal distension, abdominal pain, nausea, vomiting, and cessation of flatus and bowel movements. In severe cases, it may lead to strangulating obstruction, intestinal necrosis, or even shock and death, posing a threat to the patient’s postoperative recovery and quality of life (8).

AIO refers to abnormal adhesions between intestinal segments, between intestinal segments and intra-abdominal organs, or between intestinal segments and the abdominal wall, resulting from factors such as intra-abdominal inflammation or surgical trauma. These adhesions can lead to intestinal narrowing or obstruction, impairing the smooth passage of intestinal contents (9). Following laparoscopic surgery in AA patients, the risk of AIO may increase due to intra-abdominal inflammatory responses, surgical manipulation-induced intestinal stimulation, and alterations in the intra-abdominal environment (10). Published data suggest that AIO resulting from abdominal surgery accounts for 50–80% of all AIO cases (11). Compared with traditional open surgery, laparoscopic surgery can reduce the incidence of AIO in the medium to long term postoperatively, although it cannot entirely eliminate the risk (7). Therefore, how to effectively predict and prevent the occurrence of AIO after laparoscopic surgery in AA patients has become an urgent issue in the field of surgery. Currently, most studies on AIO after laparoscopic surgery in AA patients focus on its pathogenesis, clinical manifestations, diagnostic methods, and treatment approaches (12, 13). However, in terms of risk prediction, although some studies have attempted to assess the risk of postoperative AIO occurrence using single factors such as clinical characteristics, laboratory indicators, or imaging findings, these approaches often demonstrate limited predictive accuracy and insufficient stability (14). Therefore, constructing a risk prediction model for AIO following laparoscopic surgery in AA patients based on comprehensive multifactor analysis holds substantial clinical value for improving predictive accuracy, guiding postoperative management, and reducing the incidence of complications.

Based on this, this paper establishes a risk prediction model for AIO after laparoscopic surgery for AA, incorporating clinical characteristics and laboratory indicators, through a retrospective cohort study. This model aims to accurately estimate the probability of postoperative AIO in AA patients, thereby informing clinical decision-making regarding perioperative management strategies, reducing the incidence of postoperative complications, and improving patient outcomes.

## 2 Materials and methods

### 2.1 Ethical statement

This study was approved by the Institutional Review Board and Ethics Committee of our institution. Given the retrospective nature of this study and the exclusive use of de-identified patient data, informed consent was waived as there was no risk or adverse effect on patient care. This exemption complies with regulations and ethical guidelines related to retrospective studies.

### 2.2 Study design

This retrospective cohort study enrolled 298 AA patients who underwent laparoscopic surgery in the emergency surgery department of our hospital between January 2020 and November 2024. All patient data were obtained from the electronic medical record system and were divided into an AIO group (*n* = 32) and a non-AIO group (*n* = 266) based on whether AIO occurred postoperatively.

### 2.3 Inclusion criteria

Inclusion criteria: (1) Admitted to the emergency department with acute abdominal symptoms such as metastatic right lower abdominal pain, fever, nausea, and vomiting, and after completing a physical examination, blood routine tests, and abdominal imaging examinations, meets the diagnostic criteria in the AA Clinical Diagnostic Guidelines (15); (2) Received standard three-hole laparoscopic appendectomy (LA) within 72 h of onset; (3) Age ≥18 years, no gender restrictions; (4) Complete clinical data. Exclusion criteria: (1) Organ dysfunction of the heart, lungs, brain, liver, etc.; (2) Appendiceal abscess; (3) Laparoscopic surgery failure requiring conversion to open surgery; (4) Use of a two-port or four-port surgical technique; (5) Laparoscopic surgery combined with treatment for other conditions; (6) History of abdominal surgery; (7) Concurrent primary or acquired immunodeficiency disorders; (8) Coagulation disorders.

### 2.4 Surgical approach

A three-port technique was employed, with endotracheal intubation under general anesthesia. An incision was made at the upper edge of the umbilicus, and a 5 mm trocar was inserted to establish an artificial pneumoperitoneum, maintaining a pressure of 14 mmHg. The laparoscope was then inserted. The primary operative port was located at the 10 mm trocar site at the junction of the middle and outer thirds of the line connecting the left anterior superior iliac spine and the umbilicus. The secondary operative port is a 5 mm trocar insertion site at the midpoint of the line connecting the umbilicus and the symphysis pubis. The abdominal organs are examined, and the appendix is located along the colonic ligament. The appendix is exposed, and after dissecting the appendix vessels, they are ligated and cut. The residual ends are clamped with Hemlock clamps. Irrigate the abdominal cavity and place an abdominal drainage tube if necessary. Administer routine postoperative fluid replacement, antimicrobial therapy, and other symptomatic supportive treatments.

### 2.5 Postoperative AIO assessment and management

Retrospectively collect data on the occurrence of postoperative AIO in all patients within 6 months postoperatively through the electronic medical record system or telephone follow-up. AIO is defined as the presence of symptoms such as abdominal pain, distension, nausea, vomiting, and defecation disorders after abdominal surgery, accompanied by laboratory findings of abnormally elevated white blood cell counts, abdominal X-ray images showing “fishbone-like” changes or fluid levels in the small intestine, and highly dilated colonic loops around the abdomen (16). After confirming AIO, symptomatic supportive treatment was administered, including fasting, gastrointestinal decompression, correction of electrolyte imbalances, parenteral nutrition, and antimicrobial therapy. If there was no improvement after 48 h of conservative treatment, or if there were signs of worsening condition such as strangulating obstruction or shock, surgical intervention should be promptly initiated.

### 2.6 General data collection

All patients’ general data were retrospectively collected through the electronic medical record system or telephone follow-up results, primarily including gender, age, body mass index, and disease duration at admission. Whether antibiotics were administered preoperatively, whether the patient had hypertension, diabetes, heart disease, cerebrovascular disease, or systemic inflammatory response syndrome (SIRS) preoperatively (refer to the diagnostic criteria for SIRS in the Japanese Clinical Practice Guidelines for the Management of Sepsis and Infectious Shock 2024 (17), meeting at least two of the following criteria: (1) Body temperature >38 °C or <36 °C; (2) Heart rate >90 beats per minute; (3) Respiratory rate >20 breaths per minute or partial pressure of carbon dioxide <32 mmHg; (4) White blood cell count (WBC) >12 × 10^9^/L or <4 × 10^9^/L or immature granulocytes >10%) and other preoperative characteristics, time from admission to surgery, duration of surgery, and other surgical characteristics, presence of drainage tubes postoperatively, and pathological results (purulent, percutaneous abscess, gangrene, or perforation) and other postoperative characteristics.

### 2.7 Laboratory tests

All patients’ laboratory results within 24 h of admission were retrospectively collected via the electronic medical record system or telephone follow-up. Five milliliters of venous blood were drawn in the morning on an empty stomach, and blood tests were conducted using a fully automated hematology analyzer. The following parameters were recorded: WBC, red blood cell count (RBC), hemoglobin (HGB), platelet count (PLT), albumin (ALB), potassium ion (K^+^), sodium ion (Na^+^), chloride ion (Cl^−^), neutrophil-to-lymphocyte ratio (NLR), albumin-to-globulin ratio (AGR) levels.

### 2.8 Statistical methods

Data were analyzed using SPSS 25.0 statistical software. Count data were expressed as counts (percentages) and analyzed using the chi-square test. Continuous data that followed a normal distribution were expressed as ( $\bar{x}\pm s$ ) and analyzed using the *t*-test. For indicators showing significant differences in univariate analysis, multicollinearity analysis was performed. The variance inflation factor (VIF) was ≤10, and tolerance ≥0.1, indicating no multicollinearity issues, were subjected to binary logistic regression analysis. The significance level was set at *α* = 0.05, and a risk prediction model was established. Calibration curves and internal validation receiver operating characteristic (ROC) curves were plotted to assess the predictive performance of the model. A *p*-value <0.05 was considered statistically significant.

## 3 Results

### 3.1 Comparison of general characteristics between the AIO group and the non-AIO group

There were no statistically significant differences between the two groups in terms of gender, age, body mass index, use of antibiotics, presence of hypertension, presence of diabetes, presence of cerebrovascular disease, time from admission to surgery, duration of surgery, presence of a drainage tube postoperatively, and pathological results (*p* > 0.05). However, there were statistically significant differences in disease duration (24.32 ± 5.34 vs. 20.48 ± 5.45) hours and the presence of SIRS (17:15 vs. 20:246) (*p* < 0.05). Among these, the AIO group had a longer disease course than the non-AIO group, and a higher proportion of SIRS than the non-AIO group, with statistically significant differences (*p* < 0.05), suggesting that disease duration and the presence of SIRS may be associated with the occurrence of AIO following laparoscopic surgery for AA, as shown in Table 1.

**Table 1:** Comparison of general data between the AIO group and the non-AIO group.

Index		AIO group (*n* = 32)	Non-AIO group (*n* = 266)	*χ*^2^/*t* value	*p*-value
Admission characteristics					
Sex (*n*)	Male	19	175	0.517	0.472
	Female	13	91		
Age ( $\bar{x}\pm s$ , years)		31.36 ± 5.45	31.48 ± 5.52	0.116	0.907
Body mass index ( $\bar{x}\pm s$ , kg/m^2^)		21.87 ± 2.32	21.96 ± 2.40	0.006	0.995
Disease duration ( $\bar{x}\pm s$ , hours)		24.32 ± 5.34	20.48 ± 5.45	3.774	<0.001
Preoperative characteristics					
Preoperative antibiotic use (*n*)	Yes	22	196	0.354	0.552
	No	10	70		
Hypertension (*n*)	Yes	7	54	0.043	0.835
	No	25	212		
Diabetes (*n*)	Yes	6	48	0.010	0.922
	No	26	218		
Heart disease (*n*)	Yes	5	39	0.021	0.885
	No	27	227		
Cerebrovascular disease (*n*)	Yes	4	25	0.313	0.576
	No	28	241		
Concomitant SIRS (*n*)	Yes	17	20	54.633	<0.001
	No	15	246		
Surgical characteristics					
The time from admission to surgery ( $\bar{x}\pm s$ , hours)		35.22 ± 5.20	36.54 ± 5.35	1.374	0.171
Duration of surgery ( $\bar{x}\pm s$ , minutes)		30.56 ± 4.28	31.34 ± 4.55	0.922	0.357
Postoperative features					
Postoperative drainage (*n*)	Yes	28	226	0.146	0.702
	No	4	40		
Pathological type (*n*)	Purulent	12	104	0.031	0.861
	Periappendiceal abscess	19	165	0.085	0.770
	Gangrene or perforation	10	82	0.002	0.961

### 3.2 Comparison of laboratory test results within 24 h of admission between the two groups

There were no statistically significant differences between the two groups in terms of RBC, HGB, PLT, K^+^, Na^+^, CI^+^, and AGR levels (*p* > 0.05). However, there were statistically significant differences in WBC (13.39 ± 1.56 vs. 11.21 ± 1.92), ALB (34.92 ± 3.96 vs. 41.22 ± 4.21), and NLR (4.83 ± 0.67 vs. 4.23 ± 0.84) levels (*p* < 0.05). The AIO group had higher WBC and NLR levels and lower ALB levels than the non-AIO group, suggesting that WBC, ALB, and NLR levels may be associated with the occurrence of AIO following laparoscopic surgery for AA, as shown in Table 2.

**Table 2:** Comparison of laboratory test results within 24 h of admission between the two groups ( $\bar{x}\pm s$ ).

Index	AIO group (*n* = 32)	Non-AIO group (*n* = 266)	*χ*^2^/*t* value	*p*-value
WBC (10^9^ L^−1^)	13.39 ± 1.56	11.21 ± 1.92	6.179	<0.001
RBC (10^12^ L^−1^)	4.09 ± 0.21	4.26 ± 0.63	1.514	0.131
HGB (g L^−1^)	122.34 ± 12.26	125.54 ± 13.81	1.252	0.211
PLT (10^9^ L^−1^)	185.62 ± 18.36	191.37 ± 19.65	1.574	0.116
ALB (g L^−1^)	34.92 ± 3.96	41.22 ± 4.21	8.046	<0.001
K^+^ (mmol/L)	3.61 ± 0.36	3.92 ± 0.93	1.867	0.063
Na^+^ (mmol/L)	136.61 ± 13.66	138.39 ± 13.86	0.687	0.492
CI^+^ (mmol/L)	98.33 ± 9.83	100.54 ± 10.54	1.128	0.260
NLR	4.83 ± 0.67	4.23 ± 0.84	3.892	<0.001
AGR	1.62 ± 0.52	1.73 ± 0.53	1.111	0.267

### 3.3 Independent variable assignment table

The occurrence of AIO after AA laparoscopic surgery was used as the dependent variable, and the course of the disease, presence or absence of SIRS, WBC, ALB, and NLR were used as independent variables for assignment, as shown in Table 3.

**Table 3:** Independent variable assignment table.

Index	Variant	Assignment method
Incidence of AIO after AA laparoscopy	Implicit variable	0 = no AIO occurred, 1 = AIO occurred
Disease duration	Independent variable	Measured value
Concomitant SIRS	Independent variable	0 = No, 1 = Yes
WBC	Independent variable	Measured value
ALB	Independent variable	Measured value
NLR	Independent variable	Measured value

### 3.4 Results of collinearity analysis

Collinearity diagnosis was performed on five indicators that showed differences in disease duration, presence or absence of SIRS, WBC, ALB, and NLR. No collinearity issues were found, and these indicators could be included in the binary logistic regression analysis, as shown in Table 4.

**Table 4:** Results of collinearity analysis.

Index	Variance inflation factor values	Tolerance
Disease duration	1.010	0.990
Concomitant SIRS	1.056	0.947
WBC	1.033	0.968
ALB	1.037	0.964
NLR	1.007	0.993

### 3.5 Binary logistic regression analysis of AIO after AA laparoscopic surgery

Binary logistic regression analysis showed that disease duration (OR: 1.259; 95% CI: 1.103–1.437), presence or absence of SIRS (OR: 13.940; 95% CI: 6.075–31.985), white blood cell count (WBC) (OR: 1.953; 95% CI: 1.527–2.497), neutrophil-lymphocyte ratio (NLR) (OR: 2.710; 95% CI: 1.239–5.928) were all risk factors for AIO after AA laparoscopic surgery, while ALB (OR: 0.661; 95% CI: 0.554–0.789) was a protective factor for AIO after AA laparoscopic surgery, indicating that the aforementioned indicators are all predictive factors for AIO after AA laparoscopic surgery, as shown in Table 5.

**Table 5:** Binary logistic regression analysis of AIO after AA laparoscopy.

Index	B value	SE	Wald *χ*^2^	*p*-value	OR value	95% CI
Disease duration	0.230	0.068	11.578	0.001	1.259	1.103–1.437
SIRS	2.635	0.424	38.662	<0.001	13.940	6.075–31.985
WBC	0.669	0.126	28.407	<0.001	1.953	1.527–2.497
ALB	−0.414	0.090	21.069	<0.001	0.661	0.554–0.789
NLR	0.997	0.399	6.236	0.013	2.710	1.239–5.928

### 3.6 Construction of the nomogram model

**Figure 1:**
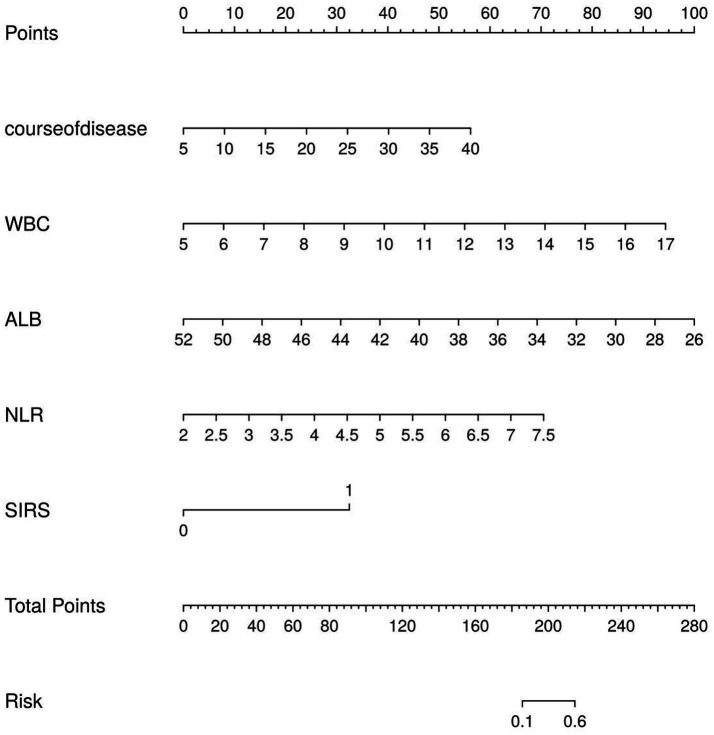
Risk prediction curve model for AIO after AA laparoscopic surgery.

Based on the results of the binary logistic regression analysis, a nomogram model for predicting the risk of AIO after AA laparoscopic surgery was constructed, as shown in Figure 1. Based on the patient’s disease course (first row), SIRS (second row), WBC (third row), ALB (fourth row), and NLR (fifth row), locate the corresponding values on the respective axes and project vertically upward to the points (score) axis to obtain the individual scores; sum the three scores and locate the corresponding position on the total points (total score) axis; Project vertically downward from the total points axis to the risk axis to obtain the probability of AA laparoscopic surgery-associated AIO occurrence.

### 3.7 Evaluation of the performance of the line chart model

**Figure 2:**
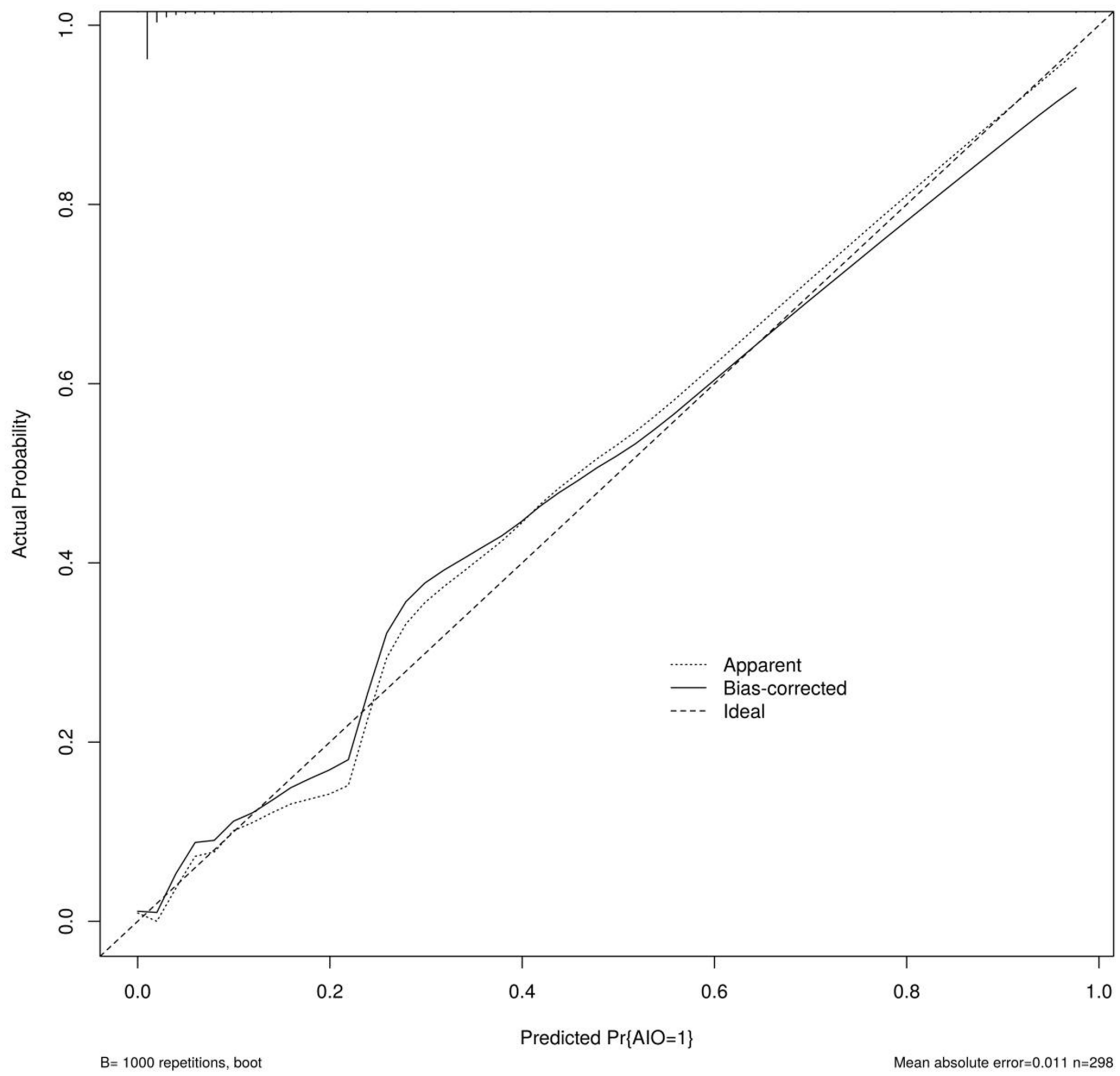
Calibration curve of the risk prediction line chart model.

**Figure 3:**
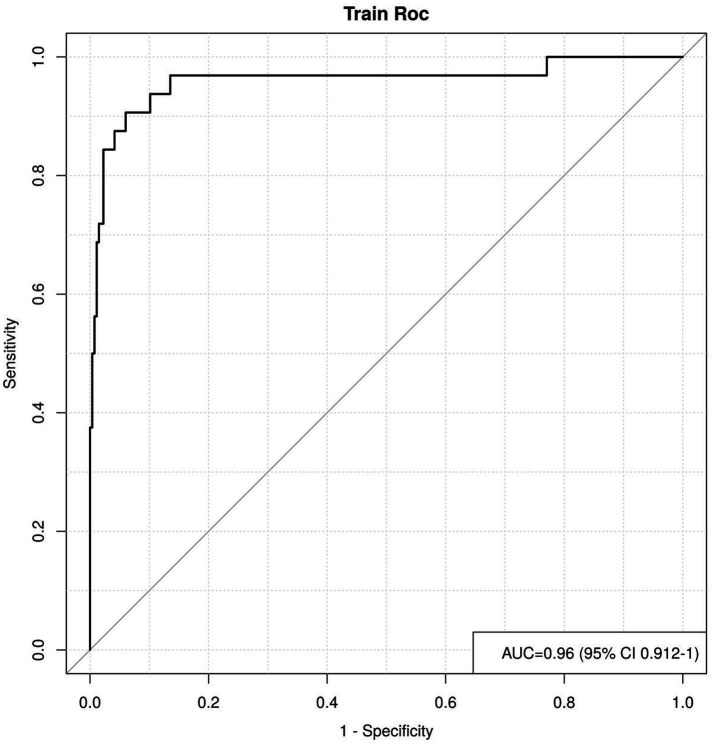
Internal validation ROC curve of the risk prediction line chart model.

Validation using bootstrap revealed that the calibration curve was close to the standard curve, indicating that the risk prediction model based on the line chart is reliable, as shown in Figure 2. The AUC of the risk prediction model based on the line chart for predicting AIO after AA laparoscopic surgery was 0.960 (95% CI: 0.912–1.000), demonstrating good discriminatory ability. This model has certain predictive value for AIO after AA laparoscopic surgery, as shown in Figure 3.

## 4 Discussion

Appendicitis is a common type of acute abdominal condition, often caused by obstruction of the appendix lumen, bacterial infection, and gastrointestinal diseases. It can occur in individuals of all age groups, and early diagnosis and timely surgical treatment are critical for clinical management (18). Compared with traditional open surgery, laparoscopic surgery is undoubtedly more acceptable to patients and their families, especially in pediatric and elderly populations (19). AIO is a recognized complication that may occur following various abdominal procedures, including appendectomy, colorectal cancer resection, and intestinal anastomosis, although its incidence after laparoscopic appendectomy is comparatively low (20). While some AIO cases may resolve with conservative management, those that fail to improve or recur often require rehospitalization and surgical intervention, imposing an additional burden on patients and increasing healthcare costs (21). This study found that disease duration, presence or absence of SIRS, WBC, ALB, and NLR all influence the occurrence of AIO after AA laparoscopic surgery. Additionally, a risk prediction model based on these indicators can assist clinicians in identifying patients with a higher risk of AIO after AA laparoscopic surgery.

This study found that the duration of illness was longer in the AIO group than in the non-AIO group, and the proportion of patients with SIRS was higher in the AIO group than in the non-AIO group. Disease duration and the presence of SIRS were identified as independent risk factors for the occurrence of AIO following laparoscopic surgery for AA. Feng et al. (22) demonstrated that disease duration is closely related to the extent of peritoneal damage in AA patients. In the early stages of the disease, the appendix is confined to lumen obstruction or inflammatory edema, and the inflammatory damage to the peritoneum is relatively mild. At this stage, surgery is not only relatively simple but also reduces the risk of postoperative abdominal infection and AIO. As the disease progresses, the extent of inflammatory damage to the peritoneum significantly worsens, characterized by the activation of inflammatory factors and fibrin exudation, making it easier for adhesions to form postoperatively and progress to AIO. Fu et al. (23) suggested that in patients with concomitant SIRS preoperatively, the body is in a state of high metabolism, high-flow circulation, and excessive inflammatory response, making it easier for infection to spread beyond its initial site and affect adjacent peritoneum and organs. When infection spreads, the peritoneal cavity is stimulated by inflammation, releasing large amounts of fibrinogen and activating it within the peritoneal cavity, further forming fibrin adhesions. Although this represents a self-protective mechanism to prevent infection spread and promote tissue repair, it can lead to adhesion formation between peritoneal surfaces, restricting gastrointestinal motility and function, and ultimately increasing the risk of AIO. Therefore, clinical attention should be highly focused on patients with prolonged disease course and concomitant SIRS. Upon admission, timely diagnosis and rapid correction of electrolyte imbalances should be performed, along with intravenous administration of broad-spectrum antibiotics and appropriate prolongation of anti-inflammatory therapy postoperatively, to reduce the incidence of AIO associated with prolonged disease duration and concomitant SIRS.

This study also found that the AIO group had higher WBC and NLR levels than the non-AIO group, and lower ALB levels than the non-AIO group. WBC and NLR were risk factors for the occurrence of AIO after AA laparoscopy, while ALB was a protective factor. ALB not only reflects nutritional status during acute illness but also participates in immune processes. Low ALB levels reduce colloid osmotic pressure, leading to tissue edema and increasing the risk of abdominal fluid accumulation and exacerbated inflammation (24). Other studies have also shown that serum ALB <3.5 g/dL (or 35.45 g/L) is a risk factor for postoperative AA complications (25). When the appendix becomes inflamed due to bacterial infection, the immune system releases inflammatory factors, which stimulate the body to produce white blood cells (WBCs) that migrate to the infected site and combat infection through phagocytosis, killing, and digestion of pathogenic microorganisms (26). Elevated WBC levels indicate activation of inflammatory factors and fibrin exudation. If the fibrinolysis process is arrested, fibrin deposition and organization occur, leading to the formation of AIO (27). NLR has the potential to predict the recovery of gastrointestinal function. The synergistic action of neutrophils and lymphocytes serves as the first line of defense to maintain intestinal homeostasis. When the intestine is damaged, immune dysregulation and cell aggregation exacerbate intestinal inflammation and motility restrictions, leading to an increased incidence of AIO (28). ALB is synthesized by hepatic parenchymal cells and accounts for 40–60% of total plasma protein. Its functions include maintaining the stability of plasma colloid osmotic pressure and binding and transporting endogenous and exogenous substances. A decrease in ALB often indicates impaired immune function, liver damage, or malnutrition (29). In AA patients, reduced ALB levels are closely associated with peritoneal inflammatory responses. Increased release of inflammatory cytokines and mediators, coupled with decreased plasma colloid osmotic pressure, disrupts the balance between fibrinogen dissolution and release by peritoneal mesothelial cells. Additionally, surgical trauma to the peritoneum and serosa further exacerbates the accumulation and adhesion of fibrinogen and fibrin in the peritoneal and intestinal spaces, ultimately leading to AIO. Clinically, it is essential to dynamically monitor patients’ laboratory indicators such as WBC, ALB, and NLR, and to reinforce anti-infective therapy and nutritional support based on test results to reduce inflammatory responses, promote peritoneal and intestinal tissue repair, and lower the risk of AIO occurrence.

In this study, a nomogram prediction model was constructed by combining various factors, transforming complex multivariate regression equations into graphical representations, thereby making abstract data visualizable and readable. Different predictive variables were used to assess the approximate probability of AIO occurrence in AA patients following laparoscopic surgery, and calibration curves were plotted to show proximity to the standard curve. The internal validation ROC curve showed an AUC of 0.960 (95% CI: 0.912–1.000), indicating that this nomogram predictive model can provide substantial support for clinical practice. By predicting based on the aforementioned factors, it can serve as a reference for early screening of high-risk groups for AIO following laparoscopic surgery in AA patients and the implementation of targeted intervention strategies.

It is worth noting that while the individual risk factors identified in this study, such as disease duration, SIRS, WBC, NLR, and ALB, have been separately investigated in previous literature, few studies have integrated these clinical characteristics and laboratory indicators into a unified, validated prediction model specifically tailored for AIO following laparoscopic appendectomy. Most prior research has focused on AIO risk after open surgery or after more extensive abdominal procedures, and single-factor analyses have demonstrated limited predictive accuracy. The present study addresses this gap by combining five independently significant predictors into a comprehensive nomogram model, achieving an AUC of 0.960, which substantially exceeds the discriminative performance reported in earlier single-factor or dual-factor prediction approaches. Furthermore, this model provides a practical, user-friendly visual tool that can be readily adopted in clinical settings to facilitate individualized risk stratification and guide targeted perioperative management for AA patients undergoing laparoscopic surgery.

This study has several limitations. First, it employed a small-sample, single-center design. A limited sample size may increase the discrepancy between sample statistics and population parameters, potentially constraining the generalizability of the findings. Additionally, the study was conducted within a specific clinical setting, and the results may not be directly applicable to other environments or populations. Furthermore, the study participants were from the same geographic region, and their demographic and socioeconomic backgrounds may exhibit considerable homogeneity, making it challenging to extrapolate the findings to a broader population. The nomogram developed in this study has not yet undergone external validation, and its broader applicability warrants further investigation. The predictive model should be regarded as an auxiliary assessment tool, and individual cases should still be evaluated comprehensively in conjunction with clinical judgment. Moreover, the follow-up conditions were limited, precluding comprehensive observation of long-term complications. Therefore, future studies should incorporate large-sample, multicenter, long-term controlled trials with external validation to further confirm the clinical applicability and enhance the representativeness and reliability of the results.

## 5 Conclusion

In this retrospective cohort study, disease duration, SIRS, WBC, ALB, and NLR were identified as significant predictors of AIO following laparoscopic surgery for AA. The risk prediction model constructed based on these clinical characteristics and laboratory indicators can assist in stratifying AIO risk in AA patients following laparoscopic surgery, guiding clinicians to implement timely and effective preventive measures and ultimately reduce the incidence of AIO.

## Data Availability

The raw data supporting the conclusions of this article will be made available by the authors, without undue reservation.
